# DNA-Free Genome Editing: Past, Present and Future

**DOI:** 10.3389/fpls.2018.01957

**Published:** 2019-01-14

**Authors:** Janina Metje-Sprink, Jochen Menz, Dominik Modrzejewski, Thorben Sprink

**Affiliations:** AG Genome Editing, Institute for Biosafety in Plant Biotechnology – Julius Kühn-Institut, Quedlinburg, Germany

**Keywords:** DNA-free, Genome Editing, RGEN, CRISPR/Cas, CRISPR/Cpf, plant, TALEN, RNPs

## Abstract

Genome Editing using engineered endonuclease (GEEN) systems rapidly took over the field of plant science and plant breeding. So far, Genome Editing techniques have been applied in more than fifty different plants; including model species like Arabidopsis; main crops like rice, maize or wheat as well as economically less important crops like strawberry, peanut and cucumber. These techniques have been used for basic research as proof-of-concept or to investigate gene functions in most of its applications. However, several market-oriented traits have been addressed including enhanced agronomic characteristics, improved food and feed quality, increased tolerance to abiotic and biotic stress and herbicide tolerance. These technologies are evolving at a tearing pace and especially the field of CRISPR based Genome Editing is advancing incredibly fast. CRISPR-Systems derived from a multitude of bacterial species are being used for targeted Gene Editing and many modifications have already been applied to the existing CRISPR-Systems such as (i) alter their protospacer adjacent motif (ii) increase their specificity (iii) alter their ability to cut DNA and (iv) fuse them with additional proteins. Besides, the classical transformation system using *Agrobacteria tumefaciens* or *Rhizobium rhizogenes*, other transformation technologies have become available and additional methods are on its way to the plant sector. Some of them are utilizing solely proteins or protein-RNA complexes for transformation, making it possible to alter the genome without the use of recombinant DNA. Due to this, it is impossible that foreign DNA is being incorporated into the host genome. In this review we will present the recent developments and techniques in the field of DNA-free Genome Editing, its advantages and pitfalls and give a perspective on technologies which might be available in the future for targeted Genome Editing in plants. Furthermore, we will discuss these techniques in the light of existing– and potential future regulations.

## Introduction

**FIGURE 1:**
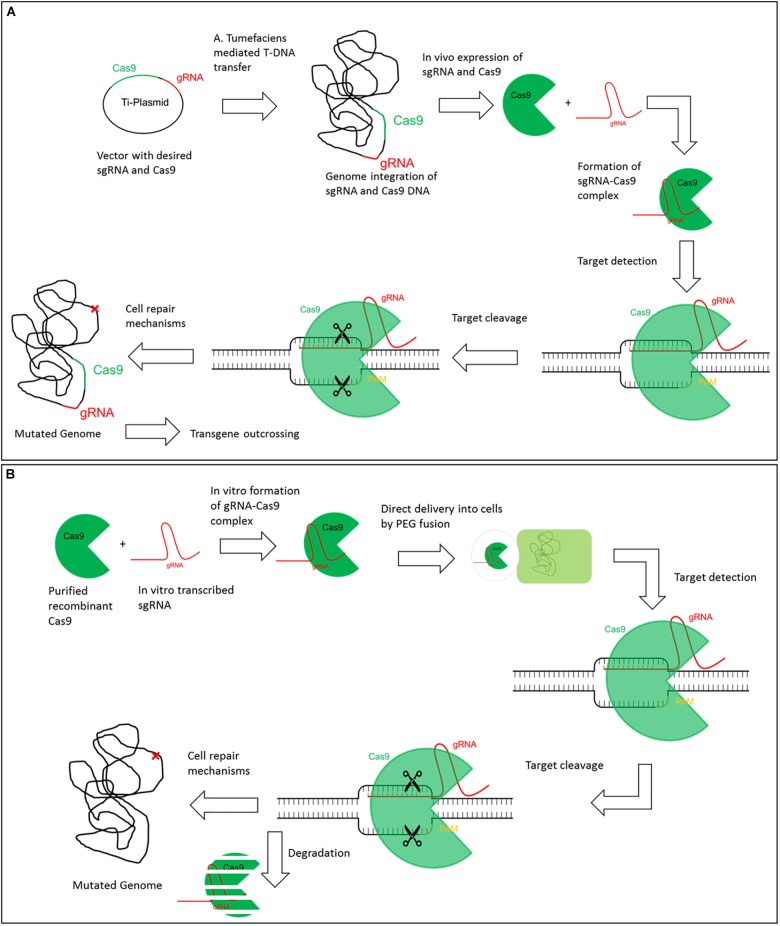
Exemplary comparison of classic CRISPR/Cas9 and DNA-free CRISPR/Cas9. Comparison of classic CRISPR/Cas9 through the example of **(A)**. tumefaciens transformation and DNA-free CRISPR/Cas9 exemplified by PEG mediated protoplast fusion. **(A)** In the classic CRISPR/Cas9 technique a T-Plasmid is designed that includes the desired gRNA and Cas9 coding sequences. Via Agrobacterium tumefaciens mediated transfer both gRNA and Cas9 sequences can be integrated in the host genome. *In vivo* gRNA and Cas9 are translated and the gRNA-Cas9 RNP complex is formed. Upon target detection, a double strand break is induced and mutations can arise by internal cell repair mechanisms. The CRISPR/Cas9 complex is constantly expressed and active in the cell. Finally, the genome can contain both the desired mutation and sequences for gRNA and Cas9. The transgene can be outcrossed but this is less practical or even impossible in vegetative propagated crops. **(B)** For DNA-free CRISPR/Cas9 recombinant Cas9 and *in vitro* translated gRNA are required. The RNP complex is formed *in vitro* and is directly delivered to protoplasts by e.g., PEG fusion. The complex is already active and can directly detect its target to induce double strand breaks. Cell repair mechanisms can lead to a mutated genome at the desired target without addition of any foreign DNA. The CRISPR/Cas9 complex is degraded within the cell and no longer available.

Genome Editing for targeted gene improvement is widely used in the field of plant science for basic research as well as for specific improvement of desired traits in commercial crops. Mainly five tools have been used for targeted Genome Editing so far. Besides Oligonucleotide Directed Mutagenesis (ODM), which had its origin in the early 80s of the last century and found its way in plant science ∼15 years ago, mainly engineered nuclease (ENs) are used (Wallace et al., 1981). There are four kinds of engineered nucleases (i) Zinc-Finger Nucleases, (ii) Meganucleases (iii) Transcription Activator Like Effector Nucleases (TALENs) and (iv) Clustered Regularly Interspaced Short Palindromic Repeats (CRISPR)-Systems. The latter is more a collection of different closely related techniques which all have been adapted for the use in Genome Editing (Puchta, 2017). Nowadays, most applications in plants (and in animals) are done by using either TALENs or CRISPR-Systems. In the majority of cases plants are stable transformed to introduce the Genome Editing tools into the plant genome (Figure 1A). Subsequently the plants are self-pollinated or crossed to get rid of the incorporated DNA, only the intended mutation remains. In some cases, transient expression of the tools e.g., via plasmids, initiate these mutations but all of these techniques make use of recombinant DNA at least in an intermediate step. Lately tools have been developed using solely RNA, preassembled Cas9 protein-gRNA ribonucleoproteins (RNPs) or TALEN-proteins for mutation induction (Figure 1B). All of these are completely free of DNA so the risk of DNA integration into the genome can be excluded. Due to this we will focus on these in the following article.

## Targeted Nucleases

Bacteria have been altering genomes since ages by using e.g., TALEs or CRISPR in combination with CRISPR associated (Cas) nucleases or other techniques such as classical restriction enzymes or Meganucleases (Roberts and Murray, 1976; Jacquier and Dujon, 1985; Stoddard, 2005; Römer et al., 2007). The aims of the bacteria using site-directed nucleases (SDNs) as tools are as diverse as ours, by using altered versions of these natural occurring mechanisms. TALEs e.g., have their origin in *Xanthomonas spec*. which manipulate cellular processes of the host by introducing TALE-proteins into plant cells via a type III secretion system (Göhre and Robatzek, 2008). Once recognized their target, TALEs alter gene expression e.g., for sugar transporters to supply the bacteria with enough resources to grow which is triggering an infection in the host such as bacterial blight (Lahaye and Bonas, 2001). Scientist revealed the hidden code of these natural occurring tracing devices and fused them with a nuclease (FokI) creating TALE-Nucleases (TALENs) (Boch et al., 2009; Cermak et al., 2011). By using them as pairs a precise induction of a DNA double strand break is possible in many organisms (Sprink et al., 2015).

CRISPR-Systems have a different origin and are ubiquitously present. Around 40% of bacteria *spec*. and 90% of archaea *spec*. sequenced so far possess one or more CRISPR-Systems (Marraffini and Sontheimer, 2010; Shmakov et al., 2017). They have been described first by Ishino et al., 1987 in the model organism *Escherichia coli* but it took additional 30 years until their function as a kind of adaptive immune system of bacteria against invading nucleic acids such as plasmids or phages have been revealed in bacteria for yogurt production (Ishino et al., 1987; Barrangou et al., 2007). Today CRISPR is still used in dairy industry to prevent phage infection in starter cultures (Grens, 2015). Additional applications have been derived from this mechanism, Jinek et al. (2012) described the ability of this technology for precise RNA guided genome modification and started the CRISPR-era (Jinek et al., 2012). Their ideas have been adopted by many scientists working in various fields and led to a new age of Genome Editing. Till now hundreds of genomes have been edited in all kinds of kingdoms and clades ranging from small viruses to trees such as Poplar (Fan et al., 2015; Yuan et al., 2015).

Besides the classical Cas9-System from *Streptococcus pyogenes* several Cas-variants from different species like *S.aureus*, *S.thermophilus* and others have been used for Genome Editing in plants (Steinert et al., 2015; Endo et al., 2016). The classical Cas9 System consists of a dual RNA-complex, CRISPR (cr) RNA and *trans* activating CRISPR (tracr) RNA. Jinek et al. (2012)., fused these two RNAs for easier cloning and handling, creating the single guide RNA (sgRNA), for which multiple vector systems are currently available.

Other systems like the CRISPR/Cpf1 (Clustered Regularly Interspaced Short Palindromic Repeats from *Prevotella and Francisella*), recently named Cas12a, differ in several aspects from the classical CRISPR/Cas-Systems as (i) the nucleases are smaller (135 vs. 158 kDa) (ii) the systems possess a natural occurring single guide RNA (iii) cutting of Cas12a results in staggered cuts Cas9 cutting in blunt (iv) the Protospacer adjacent motifs has to be rich in thymine for Cas12a and rich in guanin for Cas9 and (v) the DNA is cut distal from the recognition site by Cas12a and proximal by Cas9.

Cas13 a CRISPR-variant which is able to recognize and cut specific RNA instead of DNA has recently been exploited for RNA editing and tracking in bacteria, mammals and also plants (review Ali et al., 2018). But additional studies have to be performed to test this system for commercial applications. It offers great potential for medicine as well as for agriculture. An initial study in bacterial cells showed non-target, collateral RNA degradation, but these effects have not been reported for recent studies performed in plants and mammals (Cox et al., 2017; Aman et al., 2018).

## Current Applications

Currently in several publications’ authors promote their work as transgene-free but by taking a closer look at these publications reveal that the status of transgene-freeness focuses only on the end product. In many cases the mutation has been initiated by transient expression of plasmid based CRISPR-DNA or stable integration with subsequent backcrossing. For both techniques, integration of DNA into the host genome is still possible as plasmids are degraded in the cells and could integrate into cut sites (Woo et al., 2015). In this paper we focus on work which has been performed completely without the use of DNA for mutation initiation, meaning either RNA, RGEN RNPs or TALEN- proteins have been used for mutation induction. All of these techniques have been used successfully in plants. DNA-free editing has its origin in editing of animal cell lines or embryos where it is frequently used and is being adapted for more and more species (Hur et al., 2016; Park et al., 2017). DNA-free editing of plants is a new but emerging field which arose 2015, as concerns raised that plants transformed using DNA might be covered by gene technology laws. To date, DNA-free editing is used in at least 14 plant species, to test the ability in proof of concept studies or for improvement of yield or tolerance against biotic and abiotic stress (Table 1). The system is especially useful for species which propagate vegetative or have a long generation cycle as backcrossing is time consuming or impossible such as for potato, grapevine and apple (Table 1; Malnoy et al., 2016; Andersson et al., 2018).

**Table 1:** Recent publications using DNA-free Genome Editing approaches.

Reference	Plant species	Trait	GE-technique	Tissue	Delivery	Editing efficiency	Off-targets	Method
Woo et al., 2015	*Arabidopsis thaliana*, *Lactuca sativa*, *Nicotiana attenuata, Oryza sativa*	POC	CRISPR/Cas9 RNPs	Protoplasts	PEG-fusion	Calli: Monoallelic mutations: 5.7%; Biallelic mutations: 40%	0/104 (2–5 MM)	Targeted deep seq.
Baek et al., 2016	*Chlamydomonas reinhardtii*	Yield, abiotic stress	CRISPR/Cas9 RNPs	Single cells	Electroporation	Cells: Up to 0.56%	0/17 (2–4 MM)	Targeted deep seq.
Malnoy et al., 2016	*Malus domestica*, *Vitis vinifera*	Biotic stress	CRISPR/Cas9 RNPs	Protoplasts	PEG-fusion	Protoplasts: 0.1–6.9%	n.d.	n.d.
Stoddard et al., 2016	*Nicotiana benthamiana*	POC, herbicide resistance	TALEN mRNA	Protoplasts	PEG-fusion	Protoplasts: No-UTR DNA control: 70.5%, No-UTR mRNA control: 5.8%	n.d.	n.d.
Shin et al., 2016	*C. reinhardtii*	POC	CRISPR/Cas9 RNPs	Single cells	Electroporation	Up to 3 × 10^-5^	0/333 (1–3 MM)	WGS
Svitashev et al., 2016	Zea mays	Male sterility, herbicide tolerance	CRISPR/Cas9 RNPs	Immature embryos	Biolistics	Embryo: Cas9 only: 0.002–0.02 DNA delivery: 0.18–0.56 RNP delivery: 0.01–0.69	n.d.	n.d.
Subburaj et al., 2016	Petunia hybrid	Herbicide resistance	CRISPR/Cas9 RNPs	Protoplasts	PEG-fusion;	Protoplasts: Cas9 protein: 0.03–27.13, NR-RGEN: 5.3–34.69	n.d.	n.d.
Zhang et al., 2016	*Triticum aestivum*	Yield	CRISPR/Cas9 mRNA	immature embryos	Biolistics	No. of transgen-free frequency: 0–100% No. of homozygous transgen-free frequency: 0–36.8%	0/8 (2–4 MM)	PCR- RE
Ferenczi et al., 2017	*C. reinhardtii*	POC, Gene replacement	CRISPR/Cpf1 RNPs	Single cells	Electroporation	Colonies: 0.12–16%	n.d.	n.d.
Grahl et al., 2017	Non-albicans candida	POC	CRISPR/Cas9 RNPs	Single cells	Electroporation	n.d.	n.d.	n.d.
Kim et al., 2017	Glycine max, *N. attenuata*	Fat-synthesis; POC	CRISPR/Cpf1 RNPs	Protoplasts	PEG-fusion	Soybean: Protoplasts: LbCpf1: 0–11.7% AsCpf1: 0–1.6% Tobacco: LbCpf1 and AsCpf1: < 0.1% LbCpf1 + crRNA and AsCpf1 + crRNA: < 1%	0/23 (4–6 MM)	Targeted deep seq.
Liang et al., 2017	*T. aestivum*	Yield	CRISPR/Cas9 RNPs	Protoplasts; immature embryos	PEG fusion, Biolistics	Protoplasts: 33.4 and 21.8% Embryo cells: gw2-RNPs: 0.18–0.21% pGE-TaGW2: 0.99–1% gasr7-RNP: 45.3%	0/20 (2–5 MM)	Sanger seq.
Andersson et al., 2018	*Solanum tuberosum*	Starch syntheses	CRISPR/Cas9 RNPs	Protoplasts	PEG-fusion	Protoplasts: 1–25%	n.d.	n.d.
Liang et al., 2018	*T. aestivum*, *O. sativa*	Yield/ POC	CRISPR/Cas9/Cpf1 RNPs, TALEN-Proteins	Protoplasts; immature embryos	PEG fusion, Biolistics	Protoplasts: 10.9–33.6%	n.d.	n.d.

*GE-Technique, Genome editing technique; RNPs, Ribonucleoproteins; WGS, Whole Genome sequencing; POC, Proof of concept; n.d., not determined.*

Besides the elimination of DNA integration which circumvents the need for backcrossing and screening of the progeny, the DNA-free systems offer some additional advantages compared to the DNA-based systems as till now no off-target effects (non-target cutting) have been observed neither using targeted nor untargeted approaches for identification (Baek et al., 2016; Shin et al., 2016; Kim et al., 2017). Further advantages are that (i) it can be used without further adaption in a majority of species (even those without established genomic alteration systems) as no coding sequence or promotor have to be adapted (Grahl et al., 2017) (ii) the amount of editors can be controlled in a better way as promotor efficiency is avoided, (iii) the editors are ready to introduce mutations directly after transfection (no lagging phase). Most of these effects seems to be a result of the defined relatively short (∼48 h) persistence of the tools in the targeted organism.

But the systems also have to deal with some drawbacks as to date it is not possible to use it in all species, mainly due to undeveloped or unsuited *in vitro* techniques. Furthermore, the efficiency is lower compared to classical methods and a selection of positively edited plants is only possible by genomic selection such as sequencing. These points result in higher costs for the technique, but further optimization will result in better *in vitro* protocols and dropping costs.

## Transformation Methods

DNA-free Genome Editing is currently performed using CRISPR/Cas9 and TALENs and reagents are introduced by either transient expression of mRNAs encoding for TALENs or Cas nuclease and guide RNA or by direct delivery of isolated RNPs. When using RNPs the complex is already preassembled and active upon delivery, when using RNA, the editors have to be transcribed and the complex has to assemble which result in a short delay in activity. DNA-free transformation challenges two major problems: (i) Delivery through the plant cell wall and (ii) regeneration of plants from tissue or cell-wall free cells. To avoid the plant cell wall barrier most edits, use isolated protoplasts, single plant cells which cell wall has been enzymatic digested. Protoplasts were the first tissue which has been used for DNA-free Genome Editing as they can be targeted easily by polyethylene glycol (PEG) mediated fusion. Therefore, the RNP complex or mRNA is enclosed in PEG vesicles and fused with protoplasts. This system enables an average editing rate of around 10% which is lower compared to DNA-based systems (Svitashev et al., 2016; Andersson et al., 2018). In potato the system is efficient from the transfection to regenerated plants, it was possible to alter all four copies of a single gene in 2–3% of the regenerated shoots (Andersson et al., 2018). In other crops such as apple or grapevine the transformation system is working but regeneration protocols for edited lines are still not available as protoplast regeneration and identification of successfully modified lines is tricky and differ even between cultivars of the same species (Malnoy et al., 2016). The single-cell alga *Chlamydomonas reinhardtii* was successfully transformed with RNPs by electroporation. Although, functional protocols are available for potatoes, lettuce, tobacco, soy and petunia regeneration rate is often low (Woo et al., 2015; Subburaj et al., 2016; Kim et al., 2017). Lately also immature embryos are being used for DNA-free transformation systems. Immature embryos can be target by biolistic delivery of both RNPs and mRNA (Svitashev et al., 2016; Zhang et al., 2016; Liang et al., 2017).

More methods are available to transfer naked DNA to plants but need to be adapted to transform DNA-free Genome Editing tools. Protoplast microinjection is described since 1983 (Griesbach, 1983) and has recently been relighted for DNA delivery in oil palm (Masani et al., 2014) but have not been tested for RNP delivery so far. An optimization of biolistic delivery to plant cells where proteins are loaded into the pores of gold activated mesoporous silica nanoparticles has been described (Martin-Ortigosa and Wang, 2014) but not published for Genome Editing yet. To overcome regeneration of immature cells *in planta* particle bombardement (iPB) that targets mature plant tissue was introduced in wheat (Hamada et al., 2017). A new method for the transformation of mature plant tissue is infiltration with cell penetrating peptides (CPPs). CPPs are a class of short, positively charged peptides that can translocate across cellular membranes. Recently they have been shown to be capable of binding site-specific nucleases (Rádis-Baptista et al., 2017). Still their potential for DNA-free Genome Editing in plants needs to be exploited.

Additional methods have also been tested to porate single cells and deliver macromolecules to the cell, such as microfluidic cell deformation or sonication and furthermore such as intensive light beams are being discussed but haven’t been tested for plants so far (Han et al., 2015; Schlicher et al., 2006).

The field of DNA-free Genome Editing is still evolving and besides new delivery methods for the reagents, proteins coupled to engineered nucleases are being developed. These approaches have been tested and used in stable transformation systems but seem to be also suitable for a DNA-free approach. Besides additional Cas-systems such as Cas12a from different organism also Cas13a could be adapted for a transient RNA-editing in an DNA-free approach, leading to a transient change in expression. This is comparable to the coupling of TALEs and other activators or repressors to Cas9 to alter the expression of genes for a defined time. Furthermore, nickases are frequently used to introduce single stranded DNA breaks in plants, to enhance specificity of Cas-systems (Fauser et al., 2014). Due to the already high specificity of the DNA-free systems, nickases are not expected to be used in DNA-free approaches. Likely, other systems will be used in the near future e.g., base editors such as cytidine or adenosine deaminases, which have been used in plants already and offer great potential to be adopted for DNA-free approaches (Gaudelli et al., 2017; Zong et al., 2017). A new and highly discussed approach is to alter methylation or acetylation for Epigenome Editing, these approaches could also be used in DNA-free approaches (Maeder et al., 2013). The newest development in the field is the guidance of integrases by Cas9, to achieve a targeted recombination. This approach is still depended on integrase sites and has been tested only in yeast and mammalian cells but an intensive search for altered integrase sites is ongoing, so that in the future targeted recombination might be possible even in plants (Chaikind et al., 2016; Merrick et al., 2018).

## Regulatory Concepts and Concerns

Although several European authorities proposed ways, how to handle and interpret new plant breeding technologies in the current or an updated European legal framework (e.g., Advisory Committee on Releases to the Environment [ACRE], 2013; Swedish Board of Agriculture [SBA], 2015; Commissie Genetische Modificatie [COGEM], 2017; Federal office for consumer protection and food safety [BVL], 2017) the European court of justice (ECJ) decided fairly unscientifically on July 25th this year, that plant products derived from Genome Editing processes (other than modified by chemical or physical mutagenesis) fall under the strict regulatory framework applied for GMOs (ECLI:EU:C:2018:583)^1^. The judgment triggered strong displeasure in the European scientific community, who forecasts noticeable economic disadvantages for European plant- and seed industry (European Plant Science Organization [EPSO], 2018; European Seed association [ESA], 2018; Vlaamsche Institute Biologie [VIB], 2018). Additionally, also the Scientific advise mechanism of the European commission published a statement on the ruling in which they recall the product-based aspects of the European gene technology law and recommend “*revising the existing GMO Directive to reflect current knowledge and scientific evidence, in particular on gene editing and established techniques of genetic modification*” (The Scientific Advice Mechanism [SAM], 2018). Due to the ruling European plant breeders need to undergo expensive and time-consuming approval procedures before their products improved by GEENs can be placed on the market. In particular, for DNA-free Genome Editing approaches, this regulation is intangible due to the lacking difference to a conventionally bred plant, as no DNA from non-related crops or organisms is introduced into the plant genome and detectable, neither in the final plant product, nor in its progenies. At no time-point during generation process, the plant genome encounters foreign, recombinant DNA, which usually triggers current European GMO-regulations. The genome edits are usually indistinguishable from natural mutations (Cao et al., 2011). In addition, off-targets play a minor role in DNA-free approaches: compared with stable and transient expression, GEENs are degraded within hours and thus the GEEN’s mode of action is only present in the original cells (protoplasts) of the edited plant (Liang et al., 2015). However, as long Europe sets its focus on the generation process during approval of new plant products, also DNA-free genome edited plants will fall within the same scrutiny as the few legal GMO-plants grown in Europe. This could lead to potential trade issues and impede innovation as stated by members of the WTO lately (World trade organization [WTO], 2018).

Notwithstanding, several countries started to update their legal interpretation of GEEN. Among them are the South-American ABC: Argentina, Brazil, Chile - the United States, Canada and Israel while in Japan and Australia new regulations and a possible exemption of Genome Editing approaches from strict rulings adopted for conventional genetically modified plants are still under discussion. Giving rise to a worldwide regulatory patchwork for genome-edited plants with a diverse set of interpretations and definitions for genome-edited plants resulting in reservations between international trade partners and trade restraints between economic regions. International harmonization of regulations and definitions thus is essential to close the risk-benefit gap between precaution and innovation potential of genome edited plants (Duensing et al., 2018). Argentina pioneered with a straightforward regulation for the new Genome Editing technologies already in 2015, 2 years after the first application of CRISPR in plants. The Resolución 173/2015^2^ defines a case-by-case dependent approach, in which applicants can request the responsive authority CONABIA already during product development to determine if their products will fall under GMO regulation. Following the Cartagena protocol definitions for living modified organisms; this is only the case when the new plant product contains a **-novel-** combination of genetic material – similar to conventional transgenic approaches when a transgene is permanently detectable in the final plant product. In case of SDN-1 (NHEJ based deletion/change of a few, often less than 20 nucleotides (Lusser et al., 2011) DNA-free Genome Editing approaches act without introducing foreign DNA that would be detectable in the final plant product. SDN-2 approaches (HDR based replacement of usually less than 20 nucleotides) using a short repair DNA sequence as template, are accordingly not completely DNA-free, although in the final plant product the template is not traceable anymore. In Argentina, plant products derived from GEENs thus will become less strictly regulated than classical GMOs. Likewise, similar guidelines are expectable in Brazil and Chile, which subsequently introduced similar case-by-case, mainly product-focused regulations. Brazil for example interprets GEENs explicitly as SDN as one of several “new precision breeding innovation technologies,” which may create a product not considered a GMO in the annex I of the normative resolution no. 16/2018^3^. Recently, together with the former mentioned ABC, also Paraguay and Uruguay declared their intention to harmonize their Genome Editing-friendly regulations and to establish genome-edited plants analogous to conventionally bred plants^4^. This initiative will transform South America into a hot spot for further Genome Editing innovations. Plant products derived by GEENs still lack on the market in these countries, but it is commendable that more and more countries worldwide clarify their legal status to pave the way for the next green revolution.

## Author Contributions

TS, JM-S, and JM wrote the manuscript. DM provided and conducted the data search. JM-S provided and constructed the figure. All authors read and approved the final manuscript.

## Conflict of Interest Statement

The authors declare that the research was conducted in the absence of any commercial or financial relationships that could be construed as a potential conflict of interest.

## Footnotes

1 Ruling in the Case C-528/16: http://curia.europa.eu/juris/document/document_print.jsf?docid=204387&text=&dir=&doclang=EN&part=1&occ=first&mode=lst&pageIndex=0&cid=5226816

2 Resolución 173/2015: http://www.fao.org/faolex/results/details/en/c/LEX-FAOC144508

3 Resolution no. 16/2018: https://agrobiobrasil.org.br/wp-content/uploads/2018/05/Normative-Resolution-16-of-January-15-2018.pdf

4 XXXVI RO CAS Declaración II: http://consejocas.org/wp-content/uploads/2018/09/XXXVI-RO-CAS-Declaraci%C3%B3n-II.-T%C3%A9cnicas-de-Edici%C3%B3n-G%C3%A9nica.pdf

## References

[B1] Advisory Committee on Releases to the Environment [ACRE] (2013). *Genetically Modified Organisms: New Plant Growing Methods.* Available at: https://www.gov.uk/government/uploads/system/uploads/attachment_data/file/239542/new-techniques-used-in-plant-breeding.pdf (accessed November 20, 2018).

[B2] Ali Z. Mahas A. Mahfouz M. (2018). CRISPR/Cas13 as a tool for RNA interference. *Trends Plant Sci.* 23 374–378. 10.1016/j.tplants.2018.03.003 29605099

[B3] Aman R. Ali Z. Butt H. Mahas A. Aljedaani F. Khan M. Z. (2018). RNA virus interference via CRISPR/Cas13a system in plants. *Genome Biol.* 19:1. 10.1186/s13059-017-1381-1 29301551 PMC5755456

[B4] Andersson M. Turesson H. Olsson N. Fält A. S. Ohlsson P. Gonzalez M. N. (2018). Genome editing in potato via CRISPR-Cas9 ribonucleoprotein delivery. *Physiol. Plant.* 164 378–384. 10.1111/ppl.12731 29572864

[B5] Baek K. Kim D. H. Jeong J. Sim S. J. Melis A. Kim J.-S. (2016). DNA-free two-gene knockout in *Chlamydomonas reinhardtii* via CRISPR-Cas9 ribonucleoproteins. *Sci. Rep.* 6:30620. 10.1038/srep30620 27466170 PMC4964356

[B6] Barrangou R. Fremaux C. Deveau H. Richards M. Boyaval P. Moineau S. (2007). CRISPR provides acquired resistance against viruses in prokaryotes. *Science* 315 1709–1712. 10.1126/science.1138140 17379808

[B7] Boch J. Scholze H. Schornack S. Landgraf A. Hahn S. Kay S. (2009). Breaking the code of DNA binding specificity of TAL-type III effectors. *Science* 326 1509–1512. 10.1126/science.1178811 19933107

[B8] Cao J. Schneeberger K. Ossowski S. Günther T. Bender S. Fitz J. (2011). Whole-genome sequencing of multiple *Arabidopsis thaliana* populations. *Nat. Genet.* 43 956–963. 10.1038/ng.911 21874002

[B9] Cermak T. Doyle E. L. Christian M. Wang L. Zhang Y. Schmidt C. (2011). Efficient design and assembly of custom TALEN and other TAL effector-based constructs for DNA targeting. *Nucleic Acids Res.* 218:e82. 10.1093/nar/gkr218 21493687 PMC3130291

[B10] Chaikind B. Bessen J. L. Thompson D. B. Hu J. H. Liu D. R. (2016). A programmable Cas9-serine recombinase fusion protein that operates on DNA sequences in mammalian cells. *Nucleic Acids Res.* 44 9758–9770. 10.1093/nar/gkw707 27515511 PMC5175349

[B11] Commissie Genetische Modificatie [COGEM] (2017). *CRISPR-Cas En Gerichte Mutagenese Bij Planten.* Available at: https://www.cogem.net/showdownload.cfm?objectId=CE6A4585-9BDD-F230-0F7FBF2C1B918008&objectType=mark.hive.contentobjects.download.pdf (accessed November 20, 2018).

[B12] Cox D. B. T. Gootenberg J. S. Abudayyeh O. O. Franklin B. Kellner M. J. Joung J. (2017). RNA editing with CRISPR-Cas13. *Science* 358 1019–1027. 10.1126/science.aaq0180 29070703 PMC5793859

[B13] Duensing N. Sprink T. Parrott W. A. Fedorova M. Lema M. A. Wolt J. D. (2018). Novel features and considerations for ERA and regulation of crops produced by genome editing. *Front. Bioeng. Biotechnol.* 6:79. 10.3389/fbioe.2018.00079 29967764 PMC6016284

[B14] Endo A. Masafumi M. Kaya H. Toki S. (2016). Efficient targeted mutagenesis of rice and tobacco genomes using Cpf1 from *Francisella novicida*. *Sci. Rep.* 6:38169. 10.1038/srep38169 27905529 PMC5131344

[B15] European Plant Science Organization [EPSO] (2018). *First Reaction on the ECJ Ruling regarding mutagenesis and the Genetically Modified Organisms Directive.* Available at: http://www.epsoweb.org/webfm_send/2405 (November 20 2018).

[B16] European Seed association [ESA] (2018). *Statement on ECJ Ruling C-528/16.* Availale at: https://www.euroseeds.eu/system/files/publications/files/esa_18.0638.pdf (November 20 2018).

[B17] Fan D. Liu T. Li C. Jiao B. Li S. Hou Y. (2015). Efficient CRISPR/Cas9-mediated targeted mutagenesis in *Populus* in the first generation. *Sci. Rep.* 5:12217. 10.1038/srep12217 26193631 PMC4507398

[B18] Fauser F. Schiml S. Puchta H. (2014). Both CRISPR/Cas-based nucleases and nickases can be used efficiently for genome engineering in *Arabidopsis thaliana*. *Plant J.* 79 348–359. 10.1111/tpj.12554 24836556

[B19] Federal office for consumer protection and food safety [BVL] (2017). *Opinion on the Legal Classification of New Plant Breeding Techniques, in particular ODM and CRISPR-Cas9.* Available at: http://www.bvl.bund.de/SharedDocs/Downloads/06_Gentechnik/Opinion_on_the_legal_classification_of_New_Plant_Breeding_Techniques.pdf?__blob=publicationFile&v=2 (November 20 2018).

[B20] Ferenczi A. Pyott D. E. Xipnitou A. Molnar A. (2017). Efficient targeted DNA editing and replacement in *Chlamydomonas reinhardtii* using Cpf1 ribonucleoproteins and single-stranded DNA. *Proc. Natl. Acad. Sci. U.S.A.* 114 13567–13572. 10.1073/pnas.1710597114 29208717 PMC5754772

[B21] Gaudelli N. M. Komor A. C. Rees H. A. Packer M. S. Badran A. H. Bryson D. I. (2017). Programmable base editing of A⋅T to G⋅C in genomic DNA without DNA cleavage. *Nature* 551:464. 10.1038/nature24644 29160308 PMC5726555

[B22] Göhre V. Robatzek S. (2008). Breaking the barriers: microbial effector molecules subvert plant immunity. *Annu. Rev. Phytopathol.* 46 189–215. 10.1146/annurev.phyto.46.120407.110050 18422429

[B23] Grahl N. Demers E. G. Crocker A. W. Hogan D. A. (2017). Use of RNA-protein complexes forgenome editing in non-albicans *Candida* species. *mSphere* 2:e00218-17. 28657070 10.1128/mSphere.00218-17 PMC5480035

[B24] Grens K. (2015). There’s CRISPR in your yogurt: we’ve all been eating food enhanced by the genome-editing tool for years. *Scientist* 29 Available at: https://www.the-scientist.com/notebook/theres-crispr-in-your-yogurt-36142

[B25] Griesbach R. J. (1983). Protoplast microinjection. *Plant Mol. Biol. Rep.* 1 32–37. 10.1007/BF02712674

[B26] Hamada H. Linghu Q. Nagira Y. Miki R. Taoka N. Imai R. (2017). An in planta biolistic method for stable wheat transformation. *Sci. Rep.* 7:11443. 10.1038/s41598-017-11936-0 28904403 PMC5597576

[B27] Han X. Liu Z. Jo M. C. Zhang K. Li Y. Zeng Z. (2015). CRISPR-Cas9 delivery to hard-to-transfect cells via membrane deformation. *Sci. Adv.* 1 1–8. 10.1126/sciadv.1500454 26601238 PMC4643799

[B28] Hur J. K. Kim K. Been K. W. Baek G. Ye S. Hur J. W. (2016). Targeted mutagenesis in mice by electroporation of Cpf1 ribonucleoproteins. *Nat. Biotechnol.* 34:807. 10.1038/nbt.3596 27272385

[B29] Ishino Y. Shinagawa H. Makino K. Amemura M. Nakata A. (1987). Nucleotide sequence of the iap gene, responsible for alkaline phosphatase isozyme conversion in *Escherichia coli*, and identification of the gene product. *J. Bacteriol.* 169 5429–5433. 10.1128/jb.169.12.5429-5433.1987 3316184 PMC213968

[B30] Jacquier A. Dujon B. (1985). An intron-encoded protein is active in a gene conversion process that spreads an intron into a mitochondrial gene. *Cell* 41 383–394. 10.1016/S0092-8674(85)80011-8 3886163

[B31] Jinek M. Chylinski K. Fonfara I. Hauer M. Doudna J. A. Charpentier E. (2012). A programmable dual-RNA–guided DNA endonuclease in adaptive bacterial immunity. *Science* 337 816–821. 10.1126/science.1225829 22745249 PMC6286148

[B32] Kim H. Kim S.-T. Ryu J. Kang B.-C. Kim J.-S. Kim S.-G. (2017). CRISPR/Cpf1-mediated DNA-free plantGenome Editing. *Nat. Commun.* 8:14406. 10.1038/ncomms14406 28205546 PMC5316869

[B33] Lahaye T. Bonas U. (2001). Molecular secrets of bacterial type III effector proteins. *Trends Plant Sci.* 6 479–485. 10.1016/S1360-1385(01)02083-0 11590067

[B34] Liang X. Potter J. Kumar S. Zou Y. Quintanilla R. Sridharan M. (2015). Rapid and highly efficient mammalian cell engineering via Cas9 protein transfection. *J. Biotechnol.* 208 44–53. 10.1016/j.jbiotec.2015.04.024 26003884

[B35] Liang Z. Chen K. Li T. Zhang Y. Wang Y. Zhao Q. (2017). Efficient DNA-free genome Editing Of Bread Wheat Using CRISPR/Cas9 ribonucleoprotein complexes. *Nat. Commun.* 8:14261. 10.1038/ncomms14261 28098143 PMC5253684

[B36] Liang Z. Chen K. Zhang Y. Liu J. Yin K. Qiu J.-L. (2018). Genome editing of bread wheat using biolistic delivery of CRISPR/Cas9 in vitro transcripts or ribonucleoproteins. *Nat. Protoc.* 13:413. 10.1038/nprot.2017.145 29388938

[B37] Lusser M. Parisi C. Plan D. Rodríguez-Cerezo E. (2011). *New Plant Breeding Techniques. State-of-the-Art and Prospects for Commercial Development.* Brussels*:* Joint Research Centre.

[B38] Maeder M. L. Angstman J. F. Richardson M. E. Linder S. J. Cascio V. M. Tsai S. Q. (2013). Targeted DNA demethylation and activation of endogenous genes using programmable TALE-TET1 fusion proteins. *Nat. Biotechnol.* 31:1137. 10.1038/nbt.2726 24108092 PMC3858462

[B39] Malnoy M. Viola R. Jung M.-H. Koo O. Kim S. Kim J.-S. (2016). DNA-free genetically edited grapevine and apple protoplast using CRISPR/Cas9 ribonucleoproteins. *Front. Plant Sci.* 7:1904. 10.3389/fpls.2016.01904 28066464 PMC5170842

[B40] Marraffini L. A. Sontheimer E. J. (2010). CRISPR interference: RNA-directed adaptive immunity in bacteria and archaea. *Nat. Rev. Genet.* 11:181. 10.1038/nrg2749 20125085 PMC2928866

[B41] Martin-Ortigosa S. Wang K. (2014). Proteolistics: a biolistic method for intracellular delivery of proteins. *Trans. Res.* 23 743–756. 10.1007/s11248-014-9807-y 25092532

[B42] Masani M. Y. A. Noll G. A. Parveez G. K. A. Sambanthamurthi R. Prüfer D. (2014). Efficient transformation of oil palm protoplasts by PEG-mediated transfection and DNA microinjection. *PLoS One* 9:e96831. 10.1371/journal.pone.0096831 24821306 PMC4018445

[B43] Merrick C. A. Zhao J. Rosser S. J. (2018). Serine integrases: advancing synthetic biology. *ACS Synth. Biol.* 7 299–310. 10.1021/acssynbio.7b00308 29316791

[B44] Park K.-E. Powell A. Sandmaier S. E. S. Kim C.-M. Mileham A. Donovan D. M. (2017). Targeted gene knock-in by CRISPR/Cas ribonucleoproteins in porcine zygotes. *Sci. Rep.* 7:42458. 10.1038/srep42458 28195163 PMC5307959

[B45] Puchta H. (2017). Applying CRISPR/Cas for genome engineering in plants: the best is yet to come. *Curr. Opin. Plant Biol.* 36 1–8. 10.1016/j.pbi.2016.11.011 27914284

[B46] Rádis-Baptista G. Campelo I. S. Morlighem J. É. R. Melo L. M. Freitas V. J. (2017). Cell-penetrating peptides (CPPs): from delivery of nucleic acids and antigens to transduction of engineered nucleases for application in transgenesis. *J. Biotechnol.* 252 15–26. 10.1016/j.jbiotec.2017.05.002 28479163

[B47] Roberts R. J. Murray K. (1976). Restriction endonuclease. *CRC Crit. Rev. Biochem.* 4 123–164. 10.3109/10409237609105456 795607

[B48] Römer P. Hahn S. Jordan T. Strauß T. Bonas U. Lahaye T. (2007). Plant pathogen recognition mediated by promoter activation of the pepper Bs3 resistance gene. *Science* 318 645–648. 10.1126/science.1144958 17962564

[B49] Schlicher R. K. Radhakrishna H. Tolentino T. P. Apkarian R. P. Zarnitsyn V. Prausnitz M. R. (2006). Mechanism of intracellular delivery by acoustic cavitation. *Ultras. Med. Biol.* 32 915–924. 10.1016/j.ultrasmedbio.2006.02.1416 16785013

[B50] Shin S.-E. Lim J. M. Koh H. G. Kim E. K. Kang N. K. Jeon S. (2016). CRISPR/Cas9-induced knockout and knock-in mutations in *Chlamydomonas reinhardtii*. *Sci. Rep.* 6:27810. 10.1038/srep27810 27291619 PMC4904240

[B51] Shmakov S. Smargon A. Scott D. Cox D. Pyzocha N. Yan W. (2017). Diversity and evolution of class 2 CRISPR-Cas systems. *Nat. Rev. Microbiol.* 15 169–182. 10.1038/nrmicro.2016.184 28111461 PMC5851899

[B52] Sprink T. Metje J. Hartung F. (2015). PlantGenome editing by novel tools: TALEN and other sequence specific nucleases. *Curr. Opin. Biotechnol.* 32 47–53. 10.1016/j.copbio.2014.11.010 25448232

[B53] Steinert J. Schiml S. Fauser F. Puchta H. (2015). Highly efficient heritable plant genome engineering using Cas9 orthologues from *Streptococcus thermophilus* and *Staphylococcus aureus*. *Plant J.* 84 1295–1305. 10.1111/tpj.13078 26576927

[B54] Stoddard B. L. (2005). Homing endonuclease structure and function. *Quart. Rev. Biophys.* 38 49–95. 10.1017/S0033583505004063 16336743

[B55] Stoddard T. J. Clasen B. M. Baltes N. J. Demorest Z. L. Voytas D. F. Zhang F. (2016). Targeted mutagenesis in plant cells through transformation of sequence-specific nuclease mRNA. *PLoS One* 11:e0154634. 10.1371/journal.pone.0154634 27176769 PMC4866682

[B56] Subburaj S. Chung S. J. Lee C. Ryu S.-M. Kim D. H. Kim J.-S. (2016). Site-directed mutagenesis in *Petunia*× *hybrida* protoplast system using direct delivery of purified recombinant Cas9 ribonucleoproteins. *Plant Cell Rep.* 35 1535–1544. 10.1007/s00299-016-1937-7 26825596

[B57] Svitashev S. Schwartz C. Lenderts B. Young J. K. Cigan A. M. (2016). Genome editing in maize directed by CRISPR–Cas9 ribonucleoprotein complexes. *Nat. Commun.* 7:13274. 10.1038/ncomms13274 27848933 PMC5116081

[B58] Swedish Board of Agriculture [SBA] (2015). *CRISPR/Cas9 mutated Arabidopsis.* Available at: https://www.upsc.se/documents/Information_on_interpretation_on_CRISPR_Cas9_mutated_plants_Final.pdf (November 20 2018).

[B59] The Scientific Advice Mechanism [SAM] (2018). *Statement by the Group of Chief Scientific Advisors A Scientific Perspective on the Regulatory Status of Products Derived from Gene Editing and the Implications for the GMO Directive.* Available at: https://ec.europa.eu/info/sites/info/files/2018_11_gcsa_statement_gene_editing_2.pdf (November 20 2018).

[B60] Vlaamsche Institute Biologie [VIB] (2018). *Regulating Genome Edited Organisms as GMOs Has Negative Consequences for Agriculture, Society and Economy.* Available at: http://www.vib.be/en/news/Documents/Position%20paper%20on%20the%20ECJ%20ruling%20on%20CRISPR%2008%20Nov%202018_FINAL.pdf (November 20 2018).

[B61] Wallace R. B. Schold M. Johnson M. J. Dembek P. Itakura K. (1981). Oligonucleotide directed mutagenesis of the human β-globin gene: a general method for producing specific point mutations in cloned DNA. *Nucleic Acids Res.* 9 3647–3656. 10.1093/nar/9.15.3647 7279669 PMC327381

[B62] Woo J. W. Kim J. Kwon S. I. Corvalan C. Cho S. W. Kim H. (2015). DNA-freeGenome editing in plants with preassembled CRISPR-Cas9 ribonucleoproteins. *Nat. Biotech.* 33 1162–1164. 10.1038/nbt.3389 26479191

[B63] World trade organization [WTO] (2018). *International Statement on Agricultural Applications of Precision Biotechnology.* Available at: https://docs.wto.org/dol2fe/Pages/FE_Search/ExportFile.aspx?id=249267&filename=q/G/SPS/GEN1658R2.pdf (November 20 2018).

[B64] Yuan M. Zhang W. Wang J. Al Yaghchi C. Ahmed J. Chard L. (2015). Efficiently editing the Vaccinia virus genome using the CRISPR Cas9 system. *J. Virol.* 89 5176–5179. 10.1128/JVI.00339-15 25741005 PMC4403460

[B65] Zhang Y. Liang Z. Zong Y. Wang Y. Liu J. Chen K. (2016). Efficient and transgene-freeGenome editing in wheat through transient expression of CRISPR/Cas9 DNA or RNA. *Nat. Commun.* 7:12617. 10.1038/ncomms12617 27558837 PMC5007326

[B66] Zong Y. Wang Y. Li C. Zhang R. Chen K. Ran Y. (2017). Precise base editing in rice, wheat and maize with a Cas9-cytidine deaminase fusion. *Nat. Biotechnol.* 35 438–440. 10.1038/nbt.3811 28244994

